# The Relationship between Reversed Masked Priming and the Tri-Phasic Pattern of the Lateralised Readiness Potential

**DOI:** 10.1371/journal.pone.0093876

**Published:** 2014-04-11

**Authors:** Ellen Seiss, Marie Klippel, Christopher Hope, Frederic Boy, Petroc Sumner

**Affiliations:** 1 School of Psychology, University of Surrey, Guildford, Surrey, United Kingdom; 2 Institut für Psychologie, Humboldt-Universität zu Berlin, Berlin, Germany; 3 School of Psychology, Cardiff University, Cardiff, United Kingdom; Radboud University Nijmegen, Netherlands

## Abstract

One of the potential explanations for negative compatibility effects (NCE) in subliminal motor priming tasks has been perceptual prime-target interactions. Here, we investigate whether the characteristic tri-phasic LRP pattern associated with the NCE is caused by these prime-target interactions. We found that both the prime-related phase and the critical reversal phase remain present even on trials where the target is omitted, confirming they are elicited by the prime and mask, not by prime-target interactions. We also report that shape and size of the reversal phase are associated with response speed, consistent with a causal role for the reversal for the subsequent response latency. Additionally, we analysed sequential modulation of the NCE by previous conflicting events, even though such conflict is subliminal. In accordance with previous literature, this modulation is small but significant.

## Introduction

Behaviour can be changed by masked visual stimuli which are not perceived consciously [1]. In a typical masked priming task, a prime is presented and immediately visually masked. Afterwards a response-relevant target is displayed and the participant responds to it by pressing corresponding response keys. When the target immediately follows the prime, a positive compatibility effect (PCE) typically occurs: responses in compatible trials (prime and target stimuli are mapped to the same response) are generally faster and more accurate than responses in incompatible trials (prime and target stimuli are mapped to alternative responses). This pattern can counter-intuitively reverse when the inter-stimulus interval (ISI) increases beyond 100 ms (i.e. responses to compatible trials are slower and less accurate than responses to incompatible trials) which results in the emergence of a negative compatibility effect (NCE; Eimer & Schlaghecken, 1998).

This positive-then-negative pattern (PCE to NCE) of non-conscious priming has been associated with a tri-phasic pattern of the Lateralized Readiness Potential (LRP). The LRP is computed by subtracting event-related potentials (ERPs) of electrodes ipsilateral to the response hand from contralateral activity. This is done for each response hand separately and the difference waveforms are then averaged. The resulting LRP reflects the lateralized activity linked to the response hand, while subtracting out all non-lateralised activity. It can therefore measure lateralised covert response tendencies before the overt motor response is given [2]).

The following LRP pattern has been found in masked priming tasks with ISIs >100 ms (e.g. Eimer & Schlaghecken, 1998; [3], [4]; Jaśkowski, Białuńska, Tomanek, & Verleger, 2008; Praamstra & Seiss, 2005). An initial activation corresponding to the primed direction (prime-related activation) is followed by a ‘reversal phase’ where response tendencies appear to be opposite to the initial activation. A third phase corresponds to the response execution, which rises earlier in the incompatible compared to the compatible condition, reflecting the behavioural latency difference – the NCE. In this article we will call this phase the target-related response activation.

The aim of this paper is to test the relationship between the tri-phasic pattern of the LRP and the NCE, in the light of two types of explanation for the NCE: reversal of the motor tendency elicited by the prime and mask versus interactions between the prime and target.

### Reversal of the motor tendency

The critically interesting phase of the LRP pattern, i.e. reflecting the development of the NCE, is the reversal phase which follows initial prime-related activation, and appears to delay response activation to the target. Under three of the main accounts of the NCE, this phase is thought to represent a reversed motor tendency elicited by the prime-mask stimuli ([5]a). In these accounts, the motor representations needed to make the appropriate response are in an unfavourable state when the target information arrives. Therefore it is assumed that the occurrence of the reversal phase (i.e. the different directions of activation preceding the target-related LRP) causes the response activation in the incompatible condition to occur earlier than in the compatible condition, which in turn causes the behavioural NCE.

Here we test a shared assumption of the three 'motor reversal' accounts of the NCE that the reversed LRP phase is triggered by prime and mask *before* the target information arrives. We do not attempt to distinguish between the three accounts here, but in brief, the theories differ in whether and how motor inhibition of the initial prime-activated motor tendency is initiated, which leads to the reversal of the motor tendency. Nevertheless, we are giving a short summary of these three accounts here. Eimer and Schlaghecken (1998, 2003) attributed the reversed LRP deflection to a self-inhibitory mechanism in the motor system, which acts to suppress the initial sub-threshold motor activation evoked by the prime and provides a mechanism to keep automatic motor activation in check without the intervention of top-down control processes. Jaskowski and colleagues [6]–[8] proposed a variant of this theory, suggesting that the inhibition of the primed response tendency is triggered by another potentially relevant stimulus (i.e. the mask stimulus) that immediately follows the prime. For example, Jaśkowski et al. (2008) varied the interval of the mask with respect both to the prime and to the target while the prime-target interval remained fixed. The LRP peak of the reversal phase appeared time-locked to the mask rather than to the prime or target (for further supporting and non-supporting evidence see e.g. Boy, Clarke, & Sumner, 2008, and Sumner & Brandwood, 2008).

The “object updating”, "active mask", or “mask-induced inhibition” account proposed a third reason for reversing the response tendency, suggesting that in some circumstances the features of the mask could reverse the primed motor tendency without the need for self-inhibition in the motor system [9]–[11] Verleger, Kötter, Jaśkowski, Sprenger, & Siebner, 2006). Specifically, although a rightward prime arrow would initially cause activation of the right hand response, a mask that contained features of the primes might then prime the leftward response, so that by the time the target appears there is motor priming in the opposite direction from the prime. As the mask used in the current study does not contain features of the prime stimuli, this account is less relevant for us here (Sumner, 2008).

Please note, in all three theories, activation or inhibition of one response tendency is likely to be accompanied by the opposite effect for the response alternative, via a push-pull mechanism such as mutual inhibition (e.g. [12], [13]–[16]; [5]a). In their masked priming study, Praamstra and Seiss (2005) found evidence for this dynamic reciprocal inhibition of motor cortices during the reversal phase of the LRP. Similar activation – inhibition patterns were also reported for the initial phase in the flanker task (Verleger, Kuniecki, Möller, Fritzmannova, & Siebner, 2009).

The critical assumption based on the accounts stated above and tested in this article is that the reversal phase of the LRP is entirely produced by the prime and mask, and would therefore occur even in trials where the target is omitted. Alternatively, if the reversal phase would reflect the effect of the prime/mask on the initial stages of target processing, it would not occur if the target is absent. This basic assumption has never been tested. Relatedly, if the reversal phase is causal for the NCE, we would expect changes in its amplitude to affect response time on trials with targets. We cannot manipulate the LRP amplitude while holding stimulus factors constant, but we did test whether it covaries with response speed as predicted.

### Delayed processing through prime-target interaction

The alternative assumption, that the reversal phase is due to prime-target interactions, comes from another class of theories. These theories explain the NCE through delayed perceptual or attentional processing of targets when they share features with the primes ([17], [18],see also [19], [20]). Lleras and Enns (2005) also discussed this idea alongside their object updating theory. Adaptation-like effects such as “repetition blindness” [21], [22], whereby the system becomes less sensitive to stimuli it has just previously been exposed to, maybe ubiquitous at many levels of perceptual and cognitive processing. This can also be seen in EEG data in metacontrast masked priming studies, where the N2pc, evoked by selection of a target from two simultaneously presented stimuli, can be markedly reduced for targets presented after congruent primes (e.g. Jaśkowski, Skalska, & Verleger, 2003; Jaśkowski, Van der Lubbe, Schlotterbeck, & Verleger, 2002; Verleger & Jaśkowski, 2007; Verleger, Żurawska vel Grajewska, & Jaśkowski, 2012). Related to those habituation accounts is a proposal that the NCE emerges due to an attentional refractory period, developed as a computational model by Sohrabi & West (2008).

While a perceptual locus is not supported by some behavioural evidence [16]; Boy & Sumner, 2010; Schlaghecken & Eimer, 2004; Schlaghecken, Klapp, & Maylor, 2009), it does appear to be supported by other recent evidence provided by combining the masked prime paradigm with the PRP (psychological refractory period) paradigm (Krüger, Klapötke, & Mattler, 2011). In a dual task situation, the NCE disappeared when the interval between the stimuli for the two tasks was short, indicating - by PRP logic - that the source is before the response selection bottleneck. If response selection for the second task has to wait for the first task to complete, then differences in perceptual processing speed would be absorbed into the slack time, but differences in motor processing time would remain apparent. Also note that perceptual/attentional interactions do not require that primes and masks occupy the same location, even though co-locality has previously been seen as a requirement of perceptual theories of the NCE [23]. Many feature-specific cells in visual cortex and temporal cortex have very large receptive fields. Indeed, this was one of the motivators for Huber's (2008) model.

For our purposes, both habituation and attentional accounts share the critical factor that the NCE stems from interactions between prime and target processing outside the motor system - interactions that act to slow the perceptual processing of the target in compatible trials. These accounts do not predict that a prime/mask alone would produce reversal of the LRP. Motor effects corresponding to the NCE should only emerge once the targets are being processed. Since the reversal phase of the LRP actually occurs after target presentation, it remains theoretically possible that it depends, at least partially, on the presence of the target. The simplest way to test this is to omit the targets from some trials.

### Summary of predictions

In sum, our main question is whether the prime and mask stimuli are sufficient to induce the full reversal phase of the tri-phasic LRP, as predicted by the motor tendency accounts. Alternatively, if prime-target interactions account for the NCE the reversal phase of the LRP should not occur in the no-target condition. Of course, it could also be a combination of both accounts, reflected in a reduced magnitude of the incorrect response activation in the no-target condition. Note that it is essential to embed no-target (‘prime/mask only’) trials within blocks that contain targets on most trials in order for the primes to have associated response tendencies and produce priming at all. Our second question was whether response speed to targets covaries with the amplitude of the reversal phase, as expected if this reversal is causal for slowing or facilitating responses and thus creating the NCE. Lastly, our design affords the opportunity to analyse the effect of go and nogo trials on subliminal priming in the subsequent trial. Such sequential effects have been often reported in other conflict tasks (Gratton, Coles, & Donchin, 1992; Stürmer, Leuthold, Soetens, Schröter, & Sommer, 2002; Praamstra & Plat, 2001), and they have been explained with enhanced cognitive control after trials with conflicts (Botvinick, Braver, Barch, Carter, & Cohen, 2001). The sequential modulation of the NCE is strongly reduced in masked priming tasks where the conflict is not consciously perceived, as reported by Praamstra and Seiss (2005). It was predicted to find a similar reduction of the sequential modulation of the NCE in the current study, but it was unclear what happens to the NCE after response inhibition in the previous trial, i.e. when the previous event is a no target (nogo) trial. This research question was not the original aim of the project, but we provide the data to stimulate future research.

## Methods

### Participants

The study was conducted with 12 participants. Two were omitted from the analysis; one identified the prime even when all mask lines were presented in the staircase (ceiling effect), the other one had a poor signal-to-noise ratio in the EEG. The mean age of the remaining 10 participants (8 women) was 21.6±4.1 years. All had normal or corrected-to-normal vision and were right-handed (HQ: 0.9±0.2), except one who was ambidextrous (HQ: 0.05; Edinburgh Handedness Inventory; [24]. The study was conducted at the University of Surrey and it was approved by the University of Surrey Ethics Committee. Written informed consent was obtained from all participants for this study.

### Stimuli and experimental procedure

The stimuli were displayed in black on a white background on a 60 Hz screen 100 cm from the participant in a dimly lit room. Prime and target stimuli were left and right pointing double arrows (<< and >>, size 0.6°×0.4°). The masks were comprised of 20 randomly drawn lines on a virtual grid (0.8°×0.5°). A new mask was constructed in each trial. The fixation cross (sized 0.2°×0.2°) and the target stimuli were displayed in the screen centre, whereas the two prime and the two mask stimuli were shown at a distance of 0.8° (centre to centre measurement) to the left and right from the centre of the screen. The two simultaneously presented primes always pointed in the same direction. Responses were measured with ERTS response keys (Berisoft Cooperation, Frankfurt am Main, Germany).

The trial began with an empty screen for 300 ms, followed by a fixation cross for 1500 ms and another empty screen for 200 ms. Primes were displayed for 33 ms, immediately followed by mask stimuli shown for 100 ms. After an empty screen for 33 ms, the target occurred for 100 ms on two-thirds of the trials. On the remaining third, no target stimulus was presented ('no-target' condition). The task was to respond swiftly and accurately to the direction of the target arrow by pressing corresponding response keys with the left or right index finger. No response was required in the no-target condition. Responses were registered for 2000 ms after target onset until the prime presentation of the next trial.

Before the main experiment, participants performed a practice block of 36 trials. The main experiment consisted of 6 blocks with 120 trials. An equal number of compatible (prime and target arrows point in the same direction), incompatible (primes and target point in different directions) and no-target trials (prime and mask only) trials were presented randomly in each block.

At the end of the experiment, mask efficiency was evaluated using a staircase procedure determining the individual prime identification thresholds, as described in Seiss & Praamstra (2004). The presentation of prime and mask was identical to no-target conditions in the main experiment. Participants attempted to discriminate the direction of the prime. The number of masking lines was adapted using a fixed-step 1-up/2-down procedure, starting with an unmasked prime. Following an accurate response one random line was added to the mask; following a false response two lines were removed. The staircase lasted for 126 trials. Identification performance approaches 66% correct with this procedure [25].

### EEG data acquisition and pre-processing

The BrainVison Recorder and Analyzer (Brain Products, Munich, Germany) was used for EEG recording and analysis. The EEG was continuously DC-recorded using 32 Ag/AgCl scalp electrodes (Fp1, Fpz, Fp2, F7, F3, Fz, F4, F8, FC5, FC1, FCz, FC2, FC6, T7, T8 C3, Cz, C4, CP5, CP1, CP2, CP6, P7, P3, Pz, P4, P8, O1, Oz, O2, PO7, PO8), positioned according to the International 10–20 electrode system (American Electroencephalographic [26]). The ground electrode was AFz. Bipolar horizontal (HEOG) and vertical electro-oculogram (VEOG) derivations were used to record eye movements. The QuickAmp Signal Acquisition Device was used for the recording of the EEG and EOG signals (500 Hz sampling rate; 40 Hz low-pass filter). Offline the EEG was digitally re-referenced to the linked left and right mastoid and segmented in epochs from 100 ms before to 700 ms after prime onset. Baseline correction was applied (−100–0 ms) and trials with horizontal eye movements (Horizontal EOG threshold: ±30 μV relative to baseline), eye blinks (vertical EOG threshold: ±80 μV) or other artefacts (threshold: ±80 μV) were omitted from subsequent analysis.

For the compatible and incompatible conditions, we calculated prime-locked LRPs, where electrode sites ipsilateral to the response hand were subtracted from those contralateral to the response hand for each response hand separately, and subsequently averaged together to form the LRP. This was not possible for the no-target condition, as no response was required. Therefore, we also calculated prime-locked lateralized ERPs (L-ERPs) separately for each condition (compatible, incompatible and no-target) by subtracting the activity at electrode sites ipsilateral to the prime arrow direction (e.g. left hemisphere for left arrows) from contralateral electrode sites relative to the prime arrow direction (e.g. right hemisphere for left arrows). Subsequently, these difference waveforms were averaged. It should be noted that an LRP is a special name for an L-ERP that is recorded over motor cortical areas (C3/C4) and computed to relative to the response hand. All other potentials are called L-ERPs in the article. Please also note that the L-ERP computed relative to the prime arrow direction is the same as the LRP for the compatible condition (because the prime arrow direction, target arrow direction and response side are the same), and inversed in polarity compared to the LRP for the incompatible condition (because the prime arrow direction is opposite to the response –relevant target arrow direction).

L-ERP and LRP amplitudes were measured in the time windows of 220–270 ms (prime-related activation), 320–370 ms (reversal phase), and 470–520 ms (target-related activation) after prime onset. For the L-ERP analysis of fast vs. slow responses we added an analysis time window for the late reversal phase (370–420 ms), especially to cover the reversal phase activity for slow responses. Peak amplitudes (40 ms time window around the peak) and latencies were measured for the compatible and incompatible conditions. For the statistical analysis, amplitudes for the compatible, incompatible, no-target conditions were compared against the three corresponding baselines with two-tailed t-tests. Amplitudes and latencies were compared against each other using one way repeated measure ANOVAs with the within-participant factor Condition (compatible versus incompatible versus no-target) for the prime-related activation, reversal phase, and the target-related activation, separately.

## Results

### Behavioural Findings

**Figure 1:**
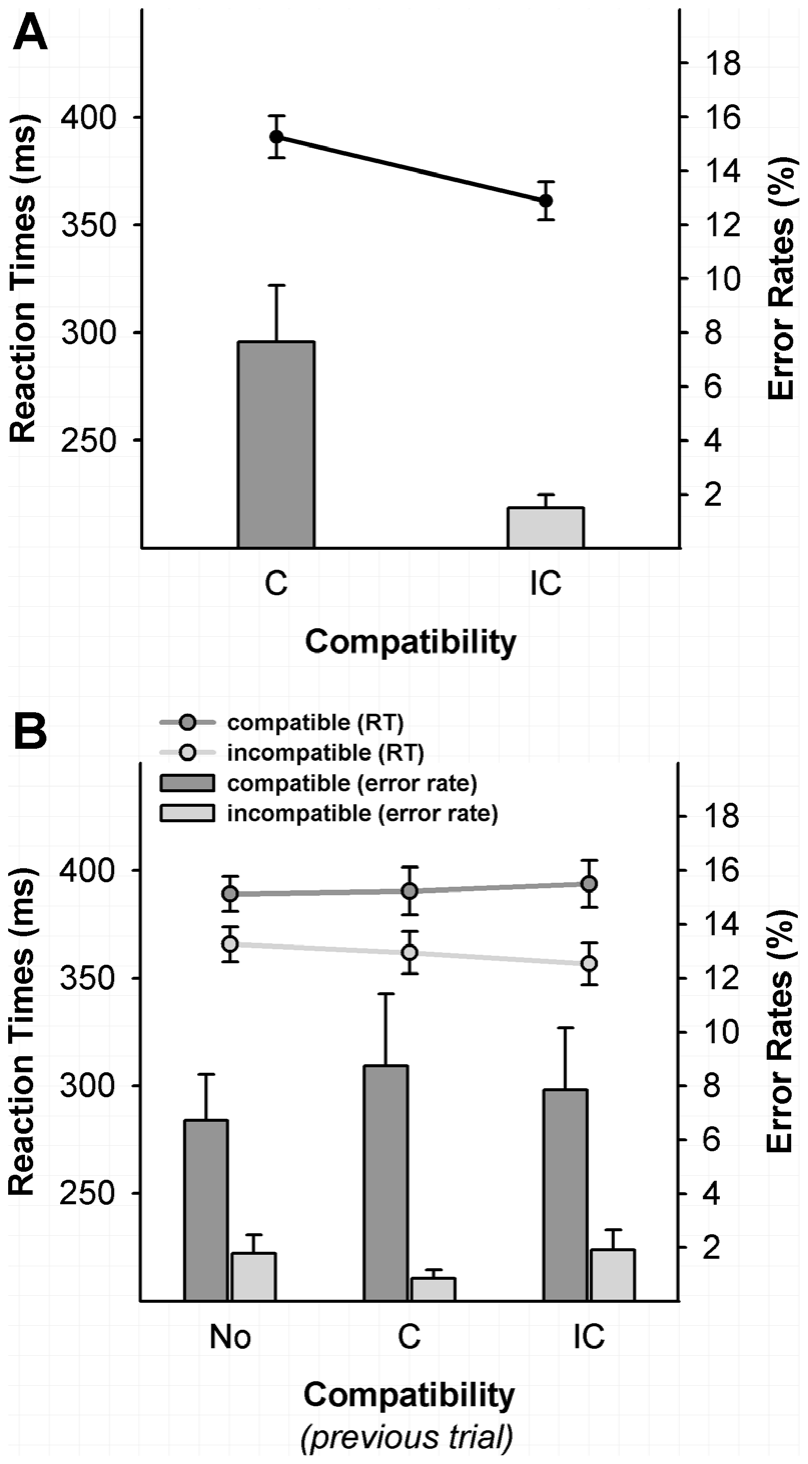
Lateralized event-related potentials (L-ERPs) for the three conditions at the electrode sites C3/C4 computed relative to (A) prime arrow direction (B) target arrow direction/response hand. (in this case the L-ERP is the same as an LRP). The upwards pointing arrow in these graphs indicates the activation in the same direction as the prime arrow/response hand (correct response). The downwards pointing arrow indicates the activation opposite to the prime arrow direction/alternative (incorrect) response hand. The grey LRP waveform represents the average of the compatible and incompatible conditions. It was added to estimate the target-related activation onset after subtracting the prime-related activation. Time scale is aligned at prime onset (0 ms). The grey bars indicate the prime-related activation, reversal phase, and target-related LRP. Displayed waveforms were additionally filtered at 12 Hz.

Mean reaction times (RTs) were only analysed for correct responses in the time window of 100–1000 ms. As shown in **Figure 1A**, RTs were significantly longer in the compatible (Mean ± SE: 390±9.9 ms) compared to the incompatible condition (361±8.9 ms; t(9) = 6.3, p<.0.001). Note that these response time effects are similar to those in previous studies with arrow targets (e.g. Eimer & Schlaghecken, 1998; Seiss & Praamstra, 2004; Praamstra & Seiss, 2005; Sumner & Brandwood, 2008; Boy et al., 2010), indicating that there has not been considerable response slowing and NCE reduction due to the intermingled nogo trials. Mean choice error rates were low (4.58±1.2%). Choice error rates were significantly higher in the compatible (7.7±2.1%) compared to the incompatible condition (1.5±0.5%, t(9) = 3.1, p = 0.012). Anticipatory responses (<100 ms) were recorded in 0.01%, late responses (>1000 ms) in 0.03%, and missed responses in 0.25% of all trials. The false alarm rate in the no-target condition was 0.6%.

In addition, we found a small, but significant, conflict modulation by previous events (**Figure 1B**). Specifically, the interaction between previous compatibility (compatible, incompatible, no-target) x current compatibility (compatible vs. incompatible) was significant for RT (F(2,18) = 6.6, p = 0.009). The size of the NCE was −23 ms when preceded by a no-target trial, −29 ms for previous compatible trials, and −37 ms for previous incompatible trials. The NCE difference between the previous no-target and the previous incongruent conditions was the largest (14 ms) and significant (t(9), 3.9, p = 0.006). The other pairwise comparisons did not reach significance (previous compatible vs. previous incompatible: 8 ms, t(9) = 1.8, p = 0.10; previous no-target vs. previous compatible: 6 ms, t(9) = 1.8, p = .093; no Bonferroni correction). No significant effects were found in the error rate analysis.

In summary, typical behavioural NCE effects were recorded for RTs and error rates in this study. In addition, there was a small, but significant, modulation of the NCE by the previous trial, which was largest when comparing the previous incompatible (no conflict) and the no-target conditions (response inhibition).

### Lateralized Event-Related Potentials (L-ERPs)

**Figure 2:**
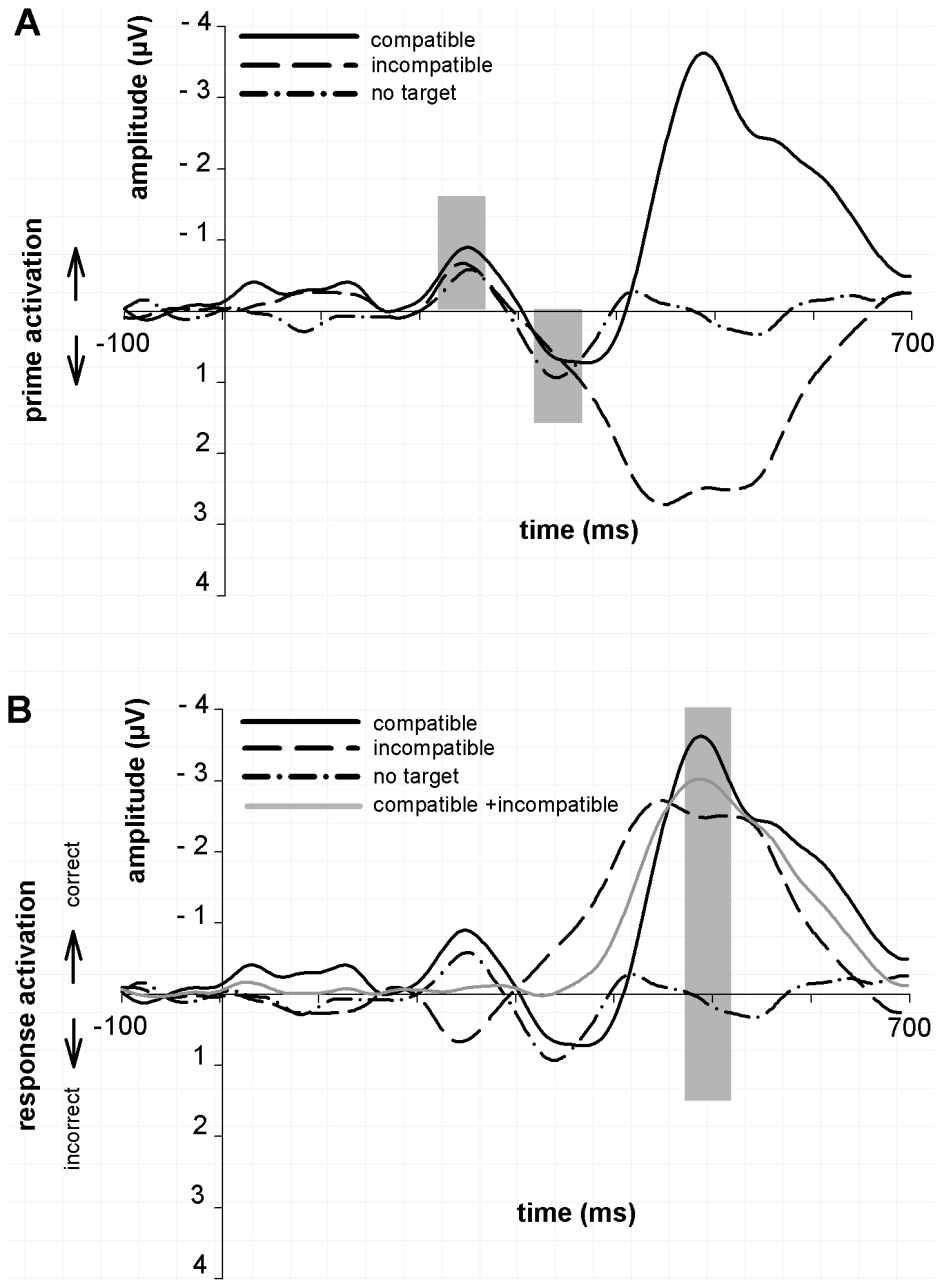
Prime-locked L-ERPs computed relative to the prime arrow direction and displayed for the three conditions at electrode pair C3/C4. L-ERPs are shown for fast responses (A) and slow responses (B). The grey bars indicate the prime-related activation, early reversal phase and late reversal phase. Displayed waveforms were filtered at 12 Hz.

The prime-locked L-ERPs for the compatible, incompatible and no-target conditions are displayed in **Figure 2**. Figure 2A shows L-ERPs that were computed by subtracting the activity of ipsi- from contralateral electrode sides relative to the prime direction (left vs. right pointing arrow). This allows a more straight forward comparison of prime-related activation and reversal phases as they have the same polarity for all three experimental conditions. Please note that the target-related response activation is of opposite-polarity for the compatible and incompatible trials, as the lateralisation for prime and target/response activation have the same direction in congruent trials, but they are of opposite polarity in incongruent trials. **Figure 2B** displays the LRPs for the compatible and incompatible conditions, calculated by subtracting the activity of ipsi- from contralateral electrode sides relative to the target direction/response hand. In this part of the figure the target-locked activation of the LRP is of the same polarity for the compatible and incompatible conditions, but the prime-related activation and reversal phase are of opposite polarity. In addition, a combined LRP was computed by averaging the compatible and incompatible conditions, in order to estimate the onset of the target-related activation, after subtracting the prime-related activation. As can be seen from Figure 2B the onset of the target-related activation in the combined LRP waveform is at about 200 ms after the target onset. This time point corresponds with the time when the activations of the congruent and incongruent condition also diverge in **Figure 2A**. Finally, the no-target L-ERP, calculated relative to the prime direction and identical to the one already displayed **Figure 2A**, was added to **Figure 2B**.

The most striking result in Figure 2 is that the no-target L-ERP appears to have, besides the initial prime-related activation, a reversal L-ERP phase comparable to the compatible and the incompatible conditions (**Figure 2A**), whereas its target-related negative activation is absent (**Figures 2A and 2B**).

#### Prime-related activation (Figure 2A)

As displayed in **Figure 2A** and confirmed by statistical analysis, mean amplitudes were significantly different from baseline for all three conditions (all t(9)>2.74, all p<0.05). However, there was no difference between the amplitudes when comparing the conditions with a one-way ANOVA, F(2,18) = 1.12, p = 0.347 (all ANOVAs were corrected for non-sphericity using the Huynh-Feldt correction).

#### Reversal Phase (Figure 2A)

Mean amplitudes were significantly different from baseline in all three conditions (all t(9)>2.65, all p<0.05) and the one-way ANOVA revealed no significant main effect of Condition, F(2,18) = 0.37, p = 0.694.

#### Target-related activation (Figure 2B)

Mean amplitudes of the compatible and incompatible condition differed significantly from baseline (all t(9)>8.08, all p<0.05). This was not the case for the no-target condition (t<1). The one-way ANOVA revealed a significant difference between all three conditions, F(2,18) = 60.13, p<0.001. The amplitude of the no-target condition was significantly reduced compared to the compatible (t = 9.48, p<0.001) and incompatible conditions (t(9) = 11.07, p<0.001). The LRP peak amplitude was significantly reduced in the incongruent compared to the congruent condition, t(9) = 3.5, p = 0.006.

The peak latency of the target-related activation was earlier in the incompatible compared to the compatible condition (Figure 2B; t(9) = 5.01, p = 0.001). The difference between incompatible and compatible LRP peak latencies also correlated with the behavioural NCE in RTs (Kendall tau, r = .67, p = .007). In sum, the target-related activation is absent in the no target condition, but present in the other two conditions. The target-locked peak latency differences between the compatible and incompatible conditions are correlated with the behavioural NCE.

#### Sequential effects

Following the small sequential effects in the behavioural NCE we analysed the L-ERPs based on previous trial type. Note that the study was not designed for this analysis, and it is rather difficult to measure L-ERP differences for effects as small as 8–14 ms, especially when the LRPs are calculated from a very small trial numbers (approx. 15–35 trials per prime arrow direction for each L-ERP/LRP waveform), but we include it as a hint for future research. There were no significant effects of previous trial on the L-ERPs, but there was a hint that prime-related activation might be smaller following a nogo trial (p = 0.14), consistent with carry over of response inhibition to the subliminal prime activity. This would be an interesting topic for further investigation.

In summary, the main finding of this subsection, and the entire study, is that the no-target L-ERP has a reversal phase comparable to the compatible and the incompatible conditions.

### Mean split of fast and slow responses: Behavioural and L-ERP data

**Figure 3:**
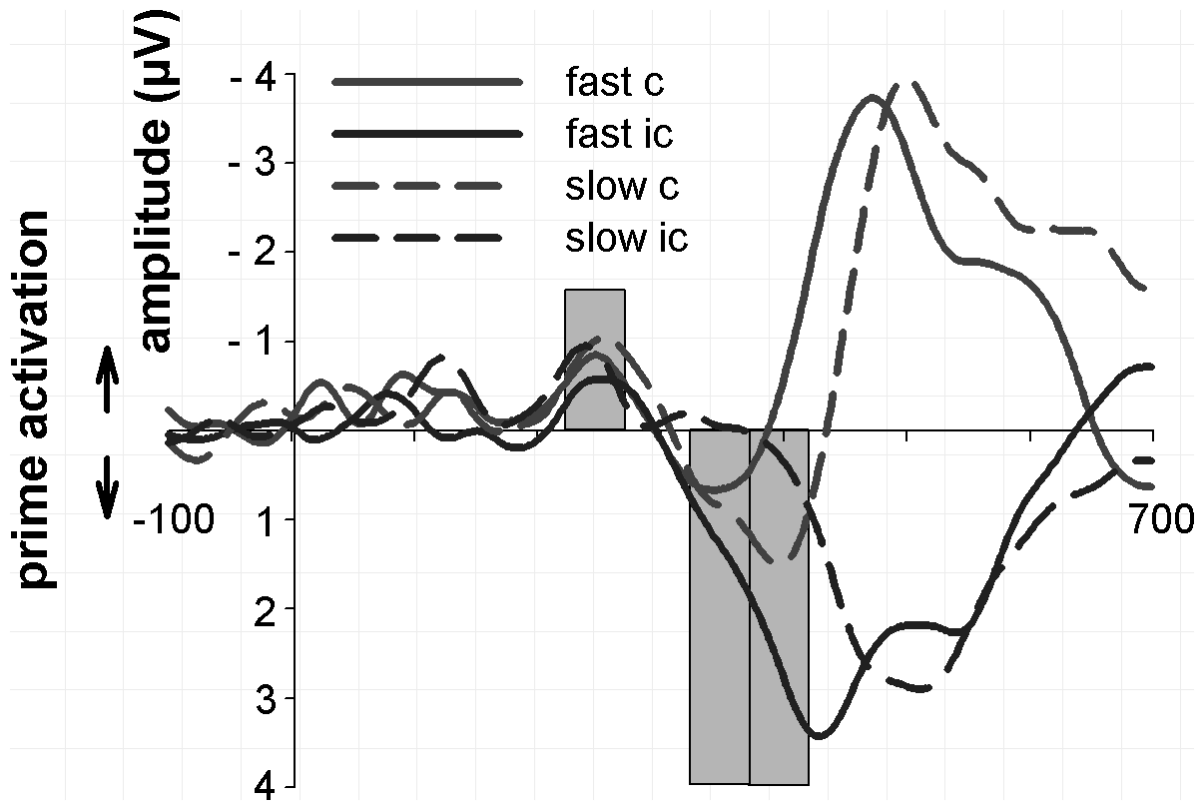
Prime-locked L-ERPs for fast and slow responses computed relative to the prime arrow direction and displayed for the compatible (red colour) and incompatible condition (blue colour) at electrode pair C3/C4. L-ERPs for fast responses are displayed as solid lines, and L-ERPs for slow responses as dashed lines. The grey bars indicate the prime-related activation, the early reversal phase, and the late reversal phase. Displayed waveforms were filtered at 12 Hz.

It has previously been assumed that the reversal phase of the LRP is causally related to the behavioural NCE, delaying responses in compatible trials and speeding them in incompatible trials (Eimer & Schlaghecken, 1998), but there is actually little evidence that directly speaks to this assumption. If this is the case, the reversal phase should be larger for *slower* compatible trials, but larger for *faster* incompatible trials. To assess this, we analysed LRPs for compatible and incompatible conditions using a mean split based on the behavioural RT (**Figure 3**).

#### Behavioural analysis

The mean reaction times for fast responses were 338±7.8 ms and for slow responses 428±11.3 ms. The fast NCE (−27±5.0 ms) was similar to the slow NCE (−28±5.5 ms), indicating that the NCE remains constant across response speeds (i.e. the 'delta plot' is on average flat, at least at the resolution of a mean split; Ridderinkhof, 2004).

#### Prime-related activation

As Figure 3 shows, the prime-related activation of the L-ERP for the compatible and incompatible condition did not differ between fast and slow responses (Compatibility x Response speed interaction: F<1).

#### Reversal phase

This picture changed for the reversal phase. In the compatible condition, the L-ERP for slow responses had a stronger reversal phase compared to the L-ERP for fast responses, as predicted. Specifically, the positive deflection was comparable in the time window between 320–370 ms (t<1), but enhanced for the slow L-ERP in the time window between 370–420 ms (t(9) = 2.9 = 0.02). The pattern was opposite for the incompatible condition, also as predicted. The incompatible L-ERP was larger for fast vs. slow responses in both time windows (320–370 ms: t(9) = 3.6, p = 0.006; 370–420 ms: t(9) = 5.6, p<0.001). For faster responses, it established quickly and was significantly different from baseline (320–370 ms: (t(9) = 3.9, p = 0.003; 370–420 ms: t(9) = 5.6, p<0.001). Its amplitude increased steadily, as it merged with the target-related activation. In contrast, the slow L-ERP did not reverse after the initial prime-related activation. It was hovering around zero (320–370 ms: t<1; 370–420 ms: t<1) before the onset of the target-related activation.

#### Target-related activation

**Figure 2** shows that the shape of the target-related activation phase of the L-ERP seems to be bimodal in the incompatible condition. **Figure 3** shows a bimodal shape with an enhanced first peak for the fast L-ERP, and a unimodal shape for the slow L-ERP. The combination of these waveforms explains the shape of the target-related activation in **Figure 2**.

Finally, peak latencies were measured and compared in an ANOVA with the factors response speed (fast vs. slow) and compatibility (compatible vs. incompatible). The main effects of speed (456±9.2 ms vs. 495±8.3 ms; F(1,9) = 44.95, p<0.001) and compatibility (c vs. ic: 489±7.7 ms vs. 463±9.9 ms; F(1,9) = 15.2, p = 0.004) were significant, but there was no significant interaction between the factors (F<1). This is consistent with the behavioural data, in which the NCE remains constant for faster and slower responses.

The main finding of this subsection is that the reversal phase was modulated by response speed. In the compatible condition, the amplitude of the reversal phase increased for slow compared to fast responses in the time window between 370–420 ms, whereas it was reduced (in fact it was absent) from the slow L-ERPs in the incompatible condition. This is consistent with the reversal phase playing a causal role in delaying compatible responses and speeding incompatible responses.

### Control measures

The efficacy of masking was measured with a staircase procedure. The thresholds, expressed as the mean number of lines required in the mask to prevent conscious perception was 13.8±3.0 lines, which is well below the number of lines (i.e. 20) used for masking in the main experiment. The primes were successfully masked and, hence, subliminal.

## Discussion

The present study examined whether a target stimulus is necessary to produce the first two deflections of the L-ERP pattern associated with the masked prime paradigm. The main findings were, firstly, a typical NCE for reaction times and error rates that was also reflected in the LRP waveform for the compatible and incompatible conditions, replicating Eimer & Schlaghecken (1998), Seiss & Praamstra (2004). Secondly, and novelly, the prime-related and reversal LRP phases were both present in the no-target condition, and of similar magnitude as in the compatible and incompatible conditions, while the target-related activation was absent as expected. Thirdly, the reversal phase of the compatible and incompatible conditions varied with response speed, in accordance with our prediction that it is causal for speeding or delaying responses. Additionally we observed a small but significant sequential modulation of the NCE by the previous trial type. These findings will be discussed in the following subsections.

### Reversal phase in the no target condition

Based on the finding that the prime-related and reversal L-ERP phases were both present in the no-target condition, and of similar magnitude as in the compatible and incompatible conditions, it can be inferred that it is not a modulation of target processing by prime-target interactions [27] that is responsible for the occurrence of the reversal L-ERP phase. Instead, the results are fully consistent with a reversal of motor tendency induced by the prime and mask, as suggested by the predominant theories of the NCE: motor self-inhibition (Eimer & Schlaghecken, 1998), object-updating (Lleras & Enns, 2004) or mask-triggered inhibition [7].

Our L-ERP results are also consistent with behavioural experiments that analysed either false alarm rates for nogo stimuli or neutral targets that required a free choice response (Schlaghecken & Eimer, 2004; Schlaghecken et al., 2009). These studies showed an increase in false alarm rates (in nogo) and a preference (in free choice) for the response that was not primed initially, implying that a motor tendency opposite to the primed direction is created by the prime and mask. Further converging behavioural data has shown that when the target-response mapping is changed during a task (for example the target that used to require a left response now requires a right response), the prime/mask stimuli continue for while to create an NCE with respect to the *previous* response mapping, not the new one (Boy & Sumner, 2010). This again implies that the prime/mask modulates response tendency rather than interacting with the current target stimulus.

### Association of reversal phase with response speed

While it has previously been assumed that the reversal phase of the LRP is causally related to the behavioural NCE, there is actually little evidence that directly speaks to this assumption. The timings of the *response*-related phase have been found to correlate with the NCE both in this study, and previously (Eimer & Schlaghecken, 1998; Boy & Sumner, unpublished result). In turn, the latency of the response phase is thought to be related to the direction and amplitude of the reversal LRP phase [28], but this assumption was not previously tested.

In order to explore this, we analysed the association of response speed with the prime-activation and reversal phases of the L-ERP. Our findings showed that the size of the behavioural NCE was not modulated by response speed, which replicates previous behavioural findings showing that the NCE does not disappear with longer RTs. The prime-related activation was also not changed by response speed. However, the reversal phase was larger for slower responses in the compatible condition, as would be predicted if the reversal phase is responsible for delaying compatible trials and creating the NCE. In incompatible trials, a normal reversal pattern was observed for fast responses, whereas the reversal phase was absent for slow responses. Again this is the direction predicted if the reversal phase is responsible for speeding incompatible responses. Of course, these are correlational results, and cannot prove cause, but they did have the potential to disprove a causal relationship if the reversal phase was not associated with response time, or the association was opposite to what we found.

Interestingly, the results imply that the balance of causes for the NCE gradually shifts from fast to slow responses. For fast responses, there are approximately equal reversal phases for compatible and incompatible trials, from which we might infer approximately equal contribution of response slowing in compatible trials and response speeding in incompatible trials. For slow responses, on the other hand, the reversal phase is absent for incompatible trials, implying that the NCE comes entirely from the extra pronounced reversal in compatible trials. To test these inferences further would need a comparison to trials with neutral primes, which this study did not include.

### Status of the perceptual accounts of the NCE

Taken together, the above-discussed results support the motor-reversal accounts of the NCE and do not support the accounts of perceptual or attentional prime-target interaction. While this is fully consistent with the working assumptions of many researchers in this area, recently the perceptual/attentional accounts had gained some ground. Previous evidence for and against perceptual accounts of the NCE has been purely behavioural. Lleras and Enns [27] found an NCE when the prime and target were presented at the same location, but a PCE when they were displayed at different locations, consistent with the prime-target interaction account where reduced repetition blindness effect is predicted for different locations. In contrast, Sumner, Tsai, Yu, & Nachev [29] reported equivalent NCEs when comparing these two conditions, and large NCEs were found in other studies where prime and targets were displayed at different locations [3_ENREF_3,4,30]. However, as mentioned in the introduction, perceptual accounts like Huber's (Huber et al., 2001, 2002) are actually quite capable of encompassing different prime and target locations because in many parts of visual cortex have large receptive fields.

Converging evidence from other studies appeared to favour response tendency modulation rather than perceptual interaction, because, as mentioned above, prime/masks can affect nogo or choice trials (Schlaghecken & Eimer, 2004; Schlaghecken et al., 2009), and they also continue to prime previous rather than current target-response mappings for several trials after a change of stimulus-response rule (see Boy & Sumner, 2010, for further explanation). On the other hand, most recently, Krüger et al. (2011) embedded the masked priming paradigm in a PRP dual task paradigm and found that the NCE disappeared when the interval between the stimuli for the two tasks was short. By PRP logic, this indicates that the source is before the response selection bottleneck (i.e. perceptual effects). However, we do not believe that this data is inconsistent with our results, because the PRP logic assumes an absolute central bottleneck where no motor activity related to the second task takes place before the bottleneck stage for the first task is complete. In contrast, we expect masked primes to automatically activate their associated motor plans immediately. If the activation of target-related response plans is then delayed due to the dual task bottleneck, the source of any motor interference, as well as any perceptual interference, from the primes could dissipate before the target responses are activated. Thus the PRP results do not uniquely support the perceptual account and can also be consistent with the assumption of a motor or response-mapping source for the NCE.

### Sequential modulation of masked priming effects

The topic of cognitive control or adaptation of behaviour based on conflicts in previous events is an interesting one, especially since subliminal primes allow us to investigate the interaction between conscious and non-conscious conflict and inhibition processes. Sequential modulations have been previously reported with other conflict tasks (Gratton et al., 1992, Stürmer et al., 2002; Praamstra & Plat, 2001; Boy et al., 2010; McBride, Sumner, & Husain, 2012b) and are explained by enhanced cognitive control after conflict trials (Botvinick et al., 2001). Praamstra and Seiss (2005) investigated the sequential modulation of the behavioural NCE with a subliminal masked priming task in order to evaluate if this modulation still occurs when the conflict is subliminal and not openly perceived (for further discussion, see McBride, et al., 2012a). They reported that the NCE modulation by a conflict in the previous event was very small in masked priming (9 ms) and strongly reduced compared to other conflict tasks where the conflicting information is visible (at least compared to the size of published effects).

In the current study, we found a similar, but non-significant, sequential modulation (8 ms) when comparing NCEs for previously presented compatible (conflict) and incompatible (no conflict) trials. Interestingly this sequential modulation was enhanced to 14 ms, and significant, when comparing the NCE for the previous incompatible (no conflict) and previous no-target events. The reduced NCE in the previous no target condition cannot be explained by a conflict between prime and target information, but can only be due to the absence of the target and the related response. This could stem from enhanced inhibition either carrying over from response inhibition in the nogo trial, or from openly visible disruption in regular sequence of events. This might be worth exploring further with other conflict tasks (e.g. systematic manipulation of the target or response presence and its relevance for the sequential modulation of conflict effects).

### Conclusions

Taken together, the present results favour the hypothesis that the reversal phase of the LRP is a reversal of motor tendency elicited by the prime and mask, and is directly related to subsequent response speed to the target. The sequential modulation of the NCE by previous conflicting events replicated previous findings by Praamstra and Seiss (2005) but also revealed that nogo trials might enhance cognitive control carrying over from response inhibition or a disruption of the regular task sequence.
